# Moderating Role of Health Literacy on the Association between Alexithymia and Depressive Symptoms in Middle School Students

**DOI:** 10.3390/ijerph17155321

**Published:** 2020-07-24

**Authors:** Xianbing Song, Danlin Li, Jie Hu, Rong Yang, Yuhui Wan, Jun Fang, Shichen Zhang

**Affiliations:** 1 Department of Human Anatomy, Histology & Embryology, Anhui Medical College, Hefei 230032, China; ayzjc2008@163.com; 2 Department of Maternal, Child and Adolescent Health, School of Public Health, Anhui Medical University, Hefei 230032, China; 15378228735@163.com (D.L.); hujie@stu.ahmu.edu.cn (J.H.); yangrong@stu.ahmu.edu.cn (R.Y.); wyhayd@163.com (Y.W.); 3 MOE Key Laboratory of Population Health Across Life Cycle/Anhui Provincial Key Laboratory of Population Health and Aristogenics, Hefei 230032, China; 4 Department of Toxicology, School of Public Health, Anhui Medical University, Hefei 230032, China; 5 Faculty of Pharmaceutical Science, Sojo University, Kumamoto 860-0082, Japan

**Keywords:** health literacy, alexithymia, depressive symptoms, moderating role, students

## Abstract

Depression is a common psychological problem in adolescents. At present, few studies have described the moderating role of health literacy on the association between alexithymia and depressive symptoms among adolescents. The purpose of this study was to explore the relation among health literacy, alexithymia, and depressive symptoms and the moderating role of health literacy in middle school students. In December 2017, data were collected from a school in Shenyang by the convenient sampling method using a questionnaire including demographic information, health literacy, alexithymia, and depressive symptoms. A total of 1068 junior and senior high school students were selected as subjects, and 1062 valid questionnaires were retained for analysis. Logistic regression models were used to examine the association between health literacy and alexithymia with depressive symptoms. The prevalence of depressive symptoms was 48.2%, and the prevalence of alexithymia was 17.9%. Low health literacy was significantly associated with depressive symptoms (odds ratio (OR) (95% confidence interval (CI)) = 3.648 (2.493–5.338)). Alexithymia was significantly correlated with depressive symptoms (OR (95% CI) = 3.091 (2.156–4.429)). Low health literacy was related to a greater increase in the risk of depressive symptoms for students with alexithymia (OR (95% CI) = 10.566 (5.175–21.570)). The findings suggest that alexithymia and health literacy are important factors influencing depressive symptoms and health literacy has a moderating role on the association between alexithymia and depressive symptoms. Enhancing health literacy of middle school students with alexithymia may improve their mental health.

## 1. Introduction

Depression is the most prevalent mental disorder around the world, but the underlying psychological cause of formation remains unclear [1]. A meta-analysis about the worldwide prevalence of mental disorders in children and adolescents showed that the prevalence of depressive disorders was 2.6% [2]. In the global burden of disease, depression is the third leading cause of disability after diarrhea and respiratory infections [3]. Notably, depressive symptoms are often formed during the adolescent period and may increase throughout adulthood [4]. Adolescence is a period that is particularly susceptible to physical and psychological factors [5]. Since the emotional regulation mechanisms of adolescents have not yet been fully developed, they are prone to have many psychological and behavioral problems [6]. So, there is no doubt that depression severely affects the health, quality of life, and well-being of adolescents [4,5,6].

Health literacy refers to the degree to which individuals have the capacity to obtain, process, and understand basic health information and services needed to make appropriate health decisions [7]. Previous studies have shown that improving health literacy can effectively improve depressive symptoms, while poor health literacy can aggravate depressive symptoms [8,9]. A key of health literacy is that it promotes conscious, strategic, and problem-solving action. Not surprisingly, adolescents who have the ability to cope with negative emotions have better mental health. In addition to being related to poor mental health, substantial evidence has indicated that poor health literacy is related to health-risk behaviors and diseases [10,11,12]. Therefore, it is reasonable to believe that adolescents with a low level of health literacy might get and use less health information and fewer health services, which may lead to more health problems [8,9,10,11,12].

Alexithymia is a cognitive–affective disturbance that is a personality trait associated with difficulties with identifying or describing one’s feelings, mental imaging and fantasy, and externally orientated cognition [13]. Studies have shown that alexithymia increases the risk of psychopathology symptoms and poor health states [14]. In fact, in most mental states, including eating disorders and anxiety, depression, alexithymia, and internal sensitivity differences are co-occurring [14,15]. Part of the reason may be that those who have alexithymia have difficulties with describing their emotions; thus, they are reluctant to tell others these problems, so that they feel helpless. However, this sense of helplessness may cause adolescents to realize that they have difficulties with social support, which may increase depressive symptoms [15]. Overall, both alexithymia and low health literacy have varying degrees of impact on adolescent depressive symptoms [8,9,10,11,12,14,15].

Nevertheless, previous studies have mostly focused on the independent association among health literacy, alexithymia, and depressive symptoms [15,16,17]. In addition to independent influence, it is possible that health literacy has a moderating role between alexithymia and depressive symptoms. Thus, this study proposes two hypotheses and examines them: (1) health literacy and alexithymia are associated with depressive symptoms, and (2) health literacy has a moderating role on the association between alexithymia and depressive symptoms in Chinese middle school students.

## 2. Methods

### 2.1. Study Participants and Procedures

We conducted this study in a boarding middle school located in Shenyang, China, by using convenient sampling in December 2017. Before investigation, we obtained informed consent from students and parents, and the investigators introduced the aims and procedures of this study to the students and assured confidentiality upon receipt of the questionnaire. If they had any questions, they could ask on the spot, and if they were not willing to participate, they could withdraw from the investigation at any time. Data were collected by a self-report questionnaire. During the investigation, all subjects were required to complete this questionnaire during a 20–30 min session in the classroom. The investigators conducted the data collection and the teacher assisted in the distribution and collection of the questionnaires. These investigators received professional training. In addition, there was no sensitive personal information in the questionnaire. After investigation, data were processed at a restricted location by using a personal, unidentifiable code for each subject.

A total of 1068 students aged 11.92–19.58 years old were recruited from grades 7–12 in the school. After discarding the invalid questionnaires (missing data > 5%), 1062 valid questionnaires were included in the analysis with the effective recovery rate 95.1%. The study was approved by the Ethics Committee of Anhui Medical University (approval number 20140087).

### 2.2. Design of Questionnaires

In the current study, the socio-demographic variables include gender, grade (junior or senior high school), registered residence area (rural or urban), any siblings, parental educational level (<high school degree or ≥high school degree), perceived family economic status (low, medium, or high), academic performance (low, medium, or high), and learning task (low, medium, or high).

The Self-Rating Depression Scale (SDS) was used to evaluate depressive symptoms in the past week [18]. The scale includes 20 questions, each of which is scored with 1 (no or little time), 2 (a little time), 3 (a lot of time), or 4 (most or all the time). The standard score ranges from 25 to 100 by summing up scores on the 20 questions and multiplying by 1.25. The score represents the level of depressive symptoms; the higher the score, the higher the level of depressive symptoms. The Chinese version of the SDS has been proven to be a reliable and effective measure in the Chinese population [19]. According to the Chinese norm of SDS, the standard score of 53 is the cut-off point [20]. That is, if a standard score is greater than or equal to 53, the person is judged to have depressive symptoms. The Cronbach’s α coefficient for the SDS was 0.770 in this study.

Health literacy was evaluated by the Chinese Adolescent Interactive Health Literacy Questionnaire (CAIHLQ), which includes 6 domains (physical activities, interpersonal relationships, stress management, self-actualization, health awareness, and dietary behavior) and 31 items. Each item was responded to with 5 answers, which were never and no desire, never but with desire, occasionally and irregularly, often, and routinely, scoring 1–5 points. The score of each item is added to get the total score, which ranges from 31 to 155. The higher scores indicate a high level of health literacy. Previous studies have demonstrated the good reliability and validity of CAIHLQ [21,22]. In the current study, the Cronbach’s α coefficient of the overall questionnaire was 0.915, and each dimension was between 0.712 and 0.843. Based on the previous studies, total scores were categorized as 3 levels: <*P*_25_, *P*_25_–*P*_75_, and >*P*_75_; the corresponding health literacy levels were low, medium, and high, respectively [21,22].

The Toronto Alexithymia Scale-20 (TAS-20) was used to assess alexithymia [23]. The scale consists of 20 items, which are scored with 1 (strongly disagree), 2 (disagree), 3 (neutrality), 4 (agree), or 5 (strongly agree), and the total score ranges from 20 to 100. According to a previous study for the TAS-20, a cut-off of 61 was set as having alexithymia [24]. The Cronbach’s α coefficient for the TAS-20 was 0.834 in our study.

### 2.3. Statistical Analysis

The chi-squared analysis was used to compare the prevalence of depressive symptoms according to group differences. Binary regression models were performed to explore the independent association of health literacy and alexithymia with depressive symptoms and the role of health literacy in the association between alexithymia and depressive symptoms. In the regression models, some key socio-demographic factors (perceived family economy status, academic performance, and learning task) were adjusted to control the influence of confounding factors. All analyses were conducted with SPSS ver. 23.0 for Windows (SPSS, Inc., Chicago, IL, USA). As usual, statistical significance was defined as *p* < 0.05.

## 3. Results

### 3.1. Characteristics of Health Literacy, Depressive Symptoms, and Alexithymia

A total of 1062 students participated in this study (576 males, 486 females). Their ages ranged from 11.92 to 19.58 years old (Mean (M) = 15.38, standard deviation (SD) = 1.74). The M ± SD score of the CAIHLQ in all participants was 106.58 ± 19.20. Descriptive statistics results showed that the values of *P*_25_ and *P*_75_ were 94 and 119, respectively. The prevalence of depressive symptoms and alexithymia was 48.2% (512/1062) and 17.9% (190/1062), respectively. Table 1 shows the prevalence of depressive symptoms by frequency characteristics. Statistical significance was found on perceived family economic status, academic performance, and learning task (*p* < 0.05 for each). In addition, a higher rate of depressive symptoms was found in those with low health literacy and alexithymia (both *p* < 0.05). There were no statistically significant differences in gender, grade, registered residence area, any siblings, or parental educational level (*p* > 0.05 for each, Table 1).

### 3.2. Binary Logistic Regression Analysis

The results in Table 2 show the independent effect of health literacy and alexithymia on depressive symptoms in the students. In the models adjusted for perceived family economic status, academic performance, and learning task, both health literacy (OR_medium_ = 1.512, 95% CI: 1.104–2.070; OR_low_ = 3.648, 95% CI: 2.493–5.338) and alexithymia (OR = 3.091, 95% CI: 2.156–4.429) remained independently associated with depressive symptoms (Table 2). Besides, the interactive term of health literacy and alexithymia had impact on depressive symptoms (*p* < 0.05, Table 2).

In addition, we examined the moderating role of health literacy on the association between alexithymia and depressive symptoms. Table 3 shows the different groups of health literacy and alexithymia with depressive symptoms. Compared with the reference group (no alexithymia and high health literacy), the crude and adjusted ORs (95% CI) of the other groups were described. The results revealed the rate of depressive symptoms decreases with increasing health literacy in groups both with and without alexithymia. After adjustment for perceived family economic status, academic performance, and learning task, the students with alexithymia and low health literacy had the highest prevalence of depressive symptoms (83.8%); OR (95% CI) value was 10.566 (5.175–21.570).

## 4. Discussion

In this study, we examined whether health literacy and alexithymia were both independently associated with depressive symptoms. Furthermore, the moderating role of health literacy on the association between alexithymia and depressive symptoms was significant, suggesting that high levels of health literacy can decrease the influence of alexithymia on depressive symptoms.

The results showed that the prevalence of depressive symptoms among middle school students was 48.2%, which is similar to a survey of four cities in China by Yu at al. (46.5%) [25]. In addition, the prevalence of depressive symptoms in our study was lower than in the Caribbean (52.1%) but higher than in Turkey (30.7%) [26,27]. However, our result was higher than another study in 2018 (25.1%) [28]. The inconsistency of these research results is related to the choice of measurement tools, the selection of the sample population, and the cultural background. The research subjects of study in 2018 were university students, but in this study, the research subjects were middle school students. According to the previous research, results indicated that middle school students have more depressive symptoms, anxiety, interpersonal problems, and other mental disorders than others [29]. This is easy to understand because middle school students have more examination and learning pressure than university students. In addition, previous studies have shown the poorer the academic performance, the higher the rate of the depressive symptoms, which is consistent with the results of this study [30]. This may be caused by the students with lower academic records having lower self-esteem and greater psychological pressure. Bad family economy means a higher rate of depressive symptoms, similar to results from a China and England longitudinal study [31]. However, in the current study, there was no correlation between depressive symptoms and gender, family structure, etc. This may be related to the fact that our subjects are from a boarding middle school, so some objective conditions such as the education method and lifestyle habits are similar and may weaken the differences caused by family structure, registered residence area, and parental educational level. Moreover, Angold et al. has explained that gender differences may not appear before the onset of puberty [32]. These differences from previous studies need to be further studied.

A characteristic of alexithymia is that it is difficult to identify and describe a person’s emotions, and the ability to accurately identify and describe a person’s emotions contributes to better mental health [33,34]. Many published studies have found that alexithymia has an adverse effect on adolescents’ mental health [35,36]. At the same time, low levels of health literacy also increase adolescents’ risk of physical and psychical problems [37,38,39]. It may be that adolescents with low levels of health literacy are not recognizing signs of negative emotion and cannot seek timely mental health services, resulting in deterioration of mental health, in line with Beck’s cognitive theory, which indicates that an individual’s negative automatic thoughts may affect emotional, physical, and motivational symptoms of depression [40]. This study also confirmed relevant conclusions that alexithymia and low levels of health literacy can increase the risk of the prevalence of depressive symptoms in adolescents independently.

To better understand the role of health literacy in the relation between alexithymia and depressive symptoms, we performed different subgroups of health literacy and alexithymia logistic regression analyses, which showed the rate of depressive symptoms among those with alexithymia was higher in those with different levels of health literacy than those without alexithymia. What is more, high levels of health literacy may decrease the risk of depressive symptoms among students with alexithymia. As we know, the cognitive function of adolescents is in the development stage, and it is often difficult for them to realize and express their emotions and the relationship between emotions and external stress. Therefore, their emotional distress often appears in the form of physical symptoms [41]. Low levels of health literacy may amplify the negative health influence of alexithymia and increase the risk of depressive symptoms among students. Simultaneously, health literacy and alexithymia may also lead to psychological problems by increasing the occurrence of bad behaviors.

The current study analyzed the association between and the moderating role of health literacy on alexithymia with depressive symptoms among middle school students. Until now, similar survey analyses have been rare, but there are still some limitations. Firstly, this study used self-assessment questionnaires, so retrospective bias was inevitable, but the questionnaires were reliable and could basically reflect the real situation. Next, the survey did not use the assessment tools of mental health literacy directly, but the CAIHLQ includes multiple dimensions such as self-actualization and stress management that were closely related to mental health literacy, and the scale has been used in many studies on psychological behavior, so the results were authentic and reliable [21,22,42,43]. Thirdly, we used a convenient sampling to investigate; only a school was surveyed, which affects the extrapolation of the results to a certain extent. Further investigations are needed to determine the validity of this study for students in other regions. Finally, this study was a cross-sectional survey, so the causal relationships among health literacy, alexithymia, and depressive symptoms of middle school students cannot be determined. It is still necessary to verify the impact of health literacy and alexithymia on the mental health of adolescents and explore the potential mechanism in a longitudinal study in the future.

## 5. Conclusions

The current study suggests that alexithymia and health literacy are important factors influencing depressive symptoms and that health literacy has a moderating role on the association between alexithymia and depressive symptoms. The study highlights that high levels of health literacy may reduce the risk of depressive symptoms in students with alexithymia. Obviously, enhancing the level of health literacy in those middle school students with alexithymia may improve their mental health.

## Figures and Tables

**Table 1:** Prevalence of depressive symptoms in the students.

Variable	Total Sample (*n* = 1062)	Depressive Symptoms		*χ^2^*	*φ/V*
		No (*n* = 550)	Yes (*n* = 512)		
Gender				0.007	0.003
Male	576 (54.2)	299 (51.9)	277 (48.1)		
Female	486 (45.8)	251 (51.6)	235 (48.4)		
Grade				0.047	−0.007
Junior school	374 (35.2)	192 (51.3)	182 (48.7)		
Senior high school	688 (64.8)	358 (52.0)	330 (48.0)		
Registered residence area				0.783	−0.027
Rural	351 (33.1)	175 (49.9)	176 (50.1)		
Urban	711 (66.9)	375 (52.7)	336 (47.3)		
Any siblings				1.310	0.035
No	635 (59.8)	338 (61.5)	297 (46.8)		
Yes	427 (40.2)	212 (49.6)	219 (50.4)		
Father’s educational level				0.031	0.005
<High school degree	563 (53.0)	293 (52.0)	270 (48.0)		
≥High school degree	499 (47.0)	257 (51.5)	242 (48.5)		
Mother’s educational level				0.854	−0.028
<High school degree	586 (55.2)	296 (50.5)	290 (49.5)		
≥High school degree	476 (44.8)	254 (53.4)	222 (46.6)		
Perceived family economy status				10.932 **	0.101 **
Low	65 (6.1)	21 (32.3)	44 (67.7)		
Medium	801 (75.4)	421 (52.6)	380 (47.4)		
High	196 (18.5)	108 (55.1)	88 (44.9)		
Perceived academic performance				29.888 ***	0.168 ***
Low	256 (24.1)	98 (38.3)	158 (61.7)		
Medium	584 (55.0)	313 (53.6)	271 (46.4)		
High	222 (20.9)	139 (62.6)	83 (37.4)		
Perceived learning task				21.275 ***	0.142 ***
Low	82 (7.7)	48 (58.5)	34 (41.5)		
Medium	667 (62.8)	374 (43.9)	293 (43.9)		
High	313 (29.5)	128 (40.9)	185 (59.1)		
Health literacy				73.244 ***	0.263 ***
Low	257 (24.2)	78 (30.4)	179 (69.6)		
Medium	524 (49.3)	285 (54.4)	239 (45.6)		
High	281 (26.5)	187 (66.5)	94 (33.5)		
Alexithymia				52.914 ***	0.223 ***
No	872 (82.1)	497 (57.0)	375 (43.0)		
Yes	190 (17.9)	53 (27.9)	137 (72.1)		

Statistical methods: chi-squared test. *φ*/*V* is effect sizes. ** *p* < 0.01; *** *p* < 0.001.

**Table 2:** The effect of health literacy and alexithymia on depressive symptoms in the students.

Variable	Depressive Symptoms		
	*n* (*%*)	Crude OR (95% CI)	Adjusted OR (95% CI) ^a^
Health literacy			
High	94 (33.5)	Ref.	Ref.
Medium	239 (45.6)	1.642 (1.207–2.234) **	1.512 (1.104–2.070) *
Low	179 (69.6)	4.208 (2.905–6.097) ***	3.648 (2.493–5.338) ***
Alexithymia			
No	375 (43.0)	Ref.	Ref.
Yes	137 (72.1)	3.111 (2.183–4.432) ***	3.091 (2.156–4.429) ***
Health literacy × Alexithymia			
High × No		Ref.	Ref.
Medium × Yes		2.468 (1.547–3.938) ***	2.513 (1.563–4.041) ***
Low × Yes		6.623 (3.428–12.797) ***	5.801 (2.970–11.330) ***

OR is odds ratio; CI is confidence interval; * *p* < 0.05; ** *p* < 0.01; *** *p* < 0.001 compared with reference; ^a^ Adjusted for perceived family economy status, academic performance, and learning task.

**Table 3:** The moderating role of health literacy on the association between alexithymia and depressive symptoms in the students.

Alexithymia	Health Literacy	Depressive Symptoms		
		*n* (%)	Crude OR (95% CI)	Adjusted OR (95% CI) ^a^
No	High	70 (28.7)	Ref.	Ref.
	Medium	183 (41.7)	1.777 (1.270–2.487) **	1.616 (1.147–2.276) **
	Low	122 (64.6)	4.526 (3.012–6.801) ***	3.935 (2.594–5.969) ***
Yes	High	24 (64.9)	4.589 (2.212–9.520) ***	4.403 (2.090–9.275) ***
	Medium	56 (65.9)	4.800 (2.833–8.133) ***	4.530 (2.650–7.743) ***
	Low	57 (83.8)	12.881 (6.380–26.004) ***	10.566 (5.175–21.570) ***

OR, odds ratio; CI, confidence interval; ** *p* < 0.01; *** *p* < 0.001 compared with reference; ^a^ Adjusted for perceived family economy status, academic performance and learning task; Cox–Snell R-squared = 0.132, Nagelkerke R-squared = 0.176.
